# Analysis of novel predictors of acute kidney injury after non-cardiac surgery: an integrative review

**DOI:** 10.1590/2175-8239-JBN-2025-0347en

**Published:** 2026-08-31

**Authors:** Anna Carolina de Rizzo Cantoni Rosa, Petherson Mendonça dos Santos, Beatriz Moreira Silva, Eduardo Cantoni Rosa, Miguel Angelo Goes

**Affiliations:** 1 Universidade Santo Amaro, São Paulo, SP, Brazil.; 2 Universidade Federal de São Paulo, Escola Paulista de Medicina, Disciplina de Nefrologia, São Paulo, SP, Brazil.; 3 Faculdade Israelita de Ciências da Saúde Albert Einstein, São Paulo, SP, Brazil.

**Keywords:** Risk Assessment, Hemoglobin, Erythrocyte Indices, Acute Kidney Injury, Postoperative Complications, Risk Factors.

## Abstract

**Introduction:**

Acute kidney injury (AKI) is a common postoperative complication (postoperative AKI, [PO-AKI]), occurring in up to 35% of non-cardiac surgery. Some biomarkers have shown value in early identification but are underused in clinical practice due to their high cost. The SPARK score and the hemoglobin-to-red cell distribution width ratio (Hb/RDW) have emerged as accessible alternatives for predicting PO-AKI.

**Objective:**

To evaluate studies on SPARK predictors and hematological biomarkers for risk stratification of PO-AKI in non-cardiac surgery.

**Methods:**

An integrative review was conducted across four databases (PubMed/MEDLINE, LILACS, Embase, and Web of Science), covering publications between 2019 and 2025 in Portuguese and English. Studies including adults undergoing non-cardiac surgery were included. The selection process was documented using a PRISMA 2020 flow diagram.

**Results:**

Six studies were included. The SPARK score showed an AUC ranging from 0.69 to 0.81 in external validations, with superior performance in modified models for specific populations. The Hb/RDW index demonstrated good accuracy (AUC 0.714; sensitivity 72.7%; specificity 70.8%) in elderly orthopedic patients, with lower values associated with increased risk of PO-AKI.

**Discussion:**

The effectiveness of SPARK depends on the population profile, highlighting the importance of external validation and contextual adjustments. Hb, RDW, and the Hb/RDW ratio appear to be promising tools to complement SPARK.

**Conclusion:**

The application of SPARK may be limited in different contexts. Simple biomarkers such as Hb, RDW, and Hb/RDW show good predictive potential and may improve the early identification of patients at risk for PO-AKI.

## INTRODUCTION

Acute Kidney Injury (AKI) is a frequent complication in the surgical setting, characterized by an abrupt reduction in kidney function over hours or days. It is diagnosed based on an increase in serum creatinine levels or a reduction in urine output, according to the KDIGO criteria^1^. Its occurrence is associated with a significant increase in morbidity and mortality, a prolonged hospital stay, and a higher risk of progression to chronic kidney disease (CKD)^2,3,4^.

Postoperative AKI (PO-AKI) accounts for up to one-third of AKI cases in hospitalized patients^5^, varying according to the type of surgery and the patients’ preexisting clinical conditions. It is more frequent after cardiac surgery; however, in non-cardiac surgery, it may occur in up to 30–35% of patients^6,7,8^.

Despite advances in understanding the pathophysiology of PO-AKI, no specific therapies are currently available to reverse established renal dysfunction, highlighting the importance of prevention and early diagnosis to mitigate or avoid its occurrence^2^.

In this context, early detection can reduce the need for renal replacement therapy, as well as hospital length of stay, mortality, and healthcare costs^9,10,11^. However, detecting PO-AKI remains a clinical challenge, since traditional biomarkers such as serum creatinine and urine output have important limitations. Creatinine may take days to reflect deterioration in kidney function, and oliguria can be influenced by transient hemodynamic factors^9^.

In this scenario, new biomarkers have been studied as alternatives for the early identification of kidney injury. Markers such as neutrophil gelatinase-associated lipocalin (NGAL), kidney injury molecule-1 (KIM-1), and the cell stress markers TIMP-2 and IGFBP-7 (NephroCheck^®^) have demonstrated predictive value for the occurrence of PO-AKI^12^. The use of these biomarkers may improve risk stratification and guide early nephroprotective interventions (“bundles”)^13^, potentially reducing AKI progression and the need for renal replacement therapy^9,14,15,16^.

Although the literature recommends the use of biomarkers at different stages of AKI, especially during the subclinical phase of kidney injury, they are still not widely adopted in clinical practice due to technical limitations and associated costs^17,18^.

In parallel, risk scores based on perioperative factors have been widely used in cardiac surgery to guide nephroprotective strategies starting in the preoperative period^19^. On the other hand, practical and well-validated tools for assessing AKI risk in non-cardiac surgery remain scarce.

To address this gap, the SPARK (Simple Postoperative AKI Risk) score was developed by researchers in South Korea to predict PO-AKI in non-cardiac surgery, combining clinical and laboratory variables to improve risk stratification^20^.

The score was derived from a large cohort study and is based exclusively on preoperative data. Eleven factors are considered: age, sex, estimated glomerular filtration rate (eGFR), albuminuria (detected by dipstick), expected duration of surgery, urgency of the procedure, presence of diabetes mellitus, use of renin–angiotensin–aldosterone system (RAAS) blockers, hypoalbuminemia, anemia, and hyponatremia. Each variable is assigned a score proportional to its statistical relevance, and the total sum classifies the patient into four risk categories. In addition to predicting AKI occurrence, the SPARK score also assesses the severity of the injury, including outcomes such as the need for dialysis or death within 90 days after surgery. Its practical application allows the early identification of patients who may benefit from intensive monitoring and preventive strategies, making it a promising tool, especially when biomarkers are not available^2^.

Furthermore, more accessible hematological markers, such as hemoglobin (Hb), red cell distribution width (RDW), and the hemoglobin-to-red cell distribution width ratio (Hb/RDW), have shown promise as predictors of PO-AKI, suggesting that alterations in tissue oxygenation and inflammatory status play an important role in the pathophysiology of kidney injury^21^.

Given this context, this integrative review aims to evaluate studies on the SPARK predictor and complete blood count–derived markers for predicting PO-AKI in patients undergoing non-cardiac surgery, analyzing their clinical applicability and identifying gaps in the available evidence.

## METHODS

### Study design

This is an integrative literature review, which allows the inclusion of studies with heterogeneous designs, enabling a broad and comprehensive synthesis of the available evidence on a given topic.

The present manuscript is part of a broader research project entitled “SPARK as a predictor of perioperative acute kidney injury in non-cardiac surgery.” The project aims to identify predictors of perioperative AKI, focusing on clinical and demographic factors. The study was approved by the Institutional Research Ethics Committee (CAAE: 67500523.1.3001.5553, August 2023).

### Registration

As this is an integrative review, prospective registration on platforms such as PROSPERO was not performed, since these registrations are primarily intended for systematic reviews. Nevertheless, methodological rigor was ensured through a structured search strategy, predefined eligibility criteria, and independent study selection.

A retrospective registration has been submitted to PROSPERO (registration number: pending at the time of submission). The protocol will be publicly available after confirmation of registration.

### Research question — PICO framework

The research question was formulated using the PICO structure (Population, Index/Intervention, Comparison, Outcomes):*“In adult patients (≥18 years) undergoing non-cardiac surgery, do the SPARK score and the Hb/RDW ratio demonstrate adequate predictive performance for PO-AKI, as measured by the AUC, sensitivity, and/or specificity, against outcomes defined by KDIGO criteria?”*, as presented in Table 1.

**Table 1:** PICO element used to structure the research question.

PICO	Operationalization
Population	Adult patients (≥18 years) undergoing elective or urgent non-cardiac surgery in any clinical setting (hospital ward, ICU, or day surgery).
Index Test	The SPARK score (Simple Postoperative AKI Risk) applied preoperatively; and/or the Hb/RDW ratio (hemoglobin-to-red cell distribution width) applied preoperatively or during the early postoperative period.
Comparison	No mandatory comparator (single-predictor studies eligible). When available: original *vs.* modified SPARK; SPARK vs. Hb/RDW; predictor vs. standard clinical assessment.
Outcomes	Primary: postoperative AKI defined according to the KDIGO criteria (serum creatinine or urine output). Secondary: critical AKI (KDIGO stage ≥2), need for renal replacement therapy, and in-hospital or 90-day mortality.

### Search strategy

The search strategy was conducted according to recommen­dations based on the PRISMA checklist^22^ using four electronic databases: PubMed/MEDLINE, LILACS (via the Virtual Health Library [VHL]), Embase, and Web of Science. The original searches were performed in January 2025, and an updated search was conducted in April 2026 following the reviewers’ recommendations. No language restriction was applied in the updated search. The reference lists of all included studies were manually screened to identify additional potentially eligible articles.

### PubMed/MEDLINE – full search string:

((“Acute Kidney Injury”[MeSH] OR “acute kidney injury”[tiab] OR “AKI”[tiab] OR “renal failure, acute”[MeSH]) AND (“Noncardiac Surgical Procedures”[MeSH] OR “non-cardiac surgery”[tiab] OR “noncardiac surgery”[tiab] OR “postoperative complications”[MeSH] OR “postoperative AKI”[tiab] OR “surgical patients”[tiab]) AND (“SPARK”[tiab] OR “Simple Postoperative AKI Risk”[tiab] OR “Hemoglobins”[MeSH Terms] OR “hemoglobin”[tiab] OR “Erythrocyte Indices”[MeSH Terms] OR “red cell distribution width”[tiab] OR “RDW”[tiab] OR “Hb/RDW”[tiab] OR “HB/RDW ratio”[tiab]))

Filters applied: publication between 2019 and 2025 (original search); no date restriction (updated search).

### LILACS/VHL – full search string:

(acute kidney injury OR AKI OR acute renal injury) AND (non-cardiac surgery OR noncardiac surgery) AND (SPARK OR hemoglobin OR hemoglobins OR RDW OR Hb/RDW OR Hb/RDW ratio OR red cell distribution width OR erythrocyte indices)

### Embase – full search string:

(‘acute kidney injury’/exp OR ‘acute kidney injury’:ti,ab,kw) AND (‘non-cardiac surgery’:ti,ab,kw OR ‘noncardiac surgery’:ti,ab,kw OR ‘postoperative complication’/exp) AND (‘SPARK score’:ti,ab,kw OR ‘hemoglobin’/exp OR ‘hemoglobins’/exp OR ‘erythrocyte indices’/exp OR ‘red cell distribution width’:ti,ab,kw OR ‘RDW’:ti,ab,kw OR ‘Hb/RDW’:ti,ab,kw)

### Web of Science – full search string:

TS=(“acute kidney injury” OR “AKI”) AND TS=(“non-cardiac surgery” OR “noncardiac surgery” OR “postoperative”) AND TS=(“SPARK” OR “hemoglobin” OR “hemoglobins” OR “red cell distribution width” OR “RDW” OR “Hb/RDW” OR “erythrocyte indices”)

### Controlled descriptors (MeSH/DeCS)

The following controlled descriptors were used: “Acute Kidney Injury” [MeSH]; “Hemoglobins” [MeSH: D006454]; “Erythrocyte Indices” [MeSH: D004910]; “Postoperative Complications” [MeSH]; “Noncardiac Surgical Procedures” [MeSH]. The Hb/RDW index is not a direct MeSH descriptor—it is a calculated ratio derived from two established descriptors: “Hemoglobins” (D006454) and “Erythrocyte Indices” (D004910), as recognized in medical indexing guidelines. The Boolean operators “AND” and “OR” were used in com­bination to refine the search results.

### Eligibility criteria

Studies were included if they met all the following criteria: (1) published between 2019 and 2025 (justification: the SPARK score was first published in 2019, marking the starting point ofthe topic); (2) conducted in human populations; (3) involving adult participants (≥18 years); (4) non-cardiac surgery; (5) predictors of interest: SPARK score and/or the Hb/RDW ratio (or its components: Hb and RDW); and (6) outcome: PO-AKI defined by KDIGO criteria.

Studies were excluded if they: (1) evaluated exclusively cardiac surgery or mixed populations without disaggregated data for non-cardiac surgery; (2) used AKI definition criteria other than those of KDIGO without the possibility of conversion; (3) were non-original articles (editorials, letters, or review articles without primary data); or (4) involved animal models or *in vitro* studies.

### Study selection

**Figure 1:**
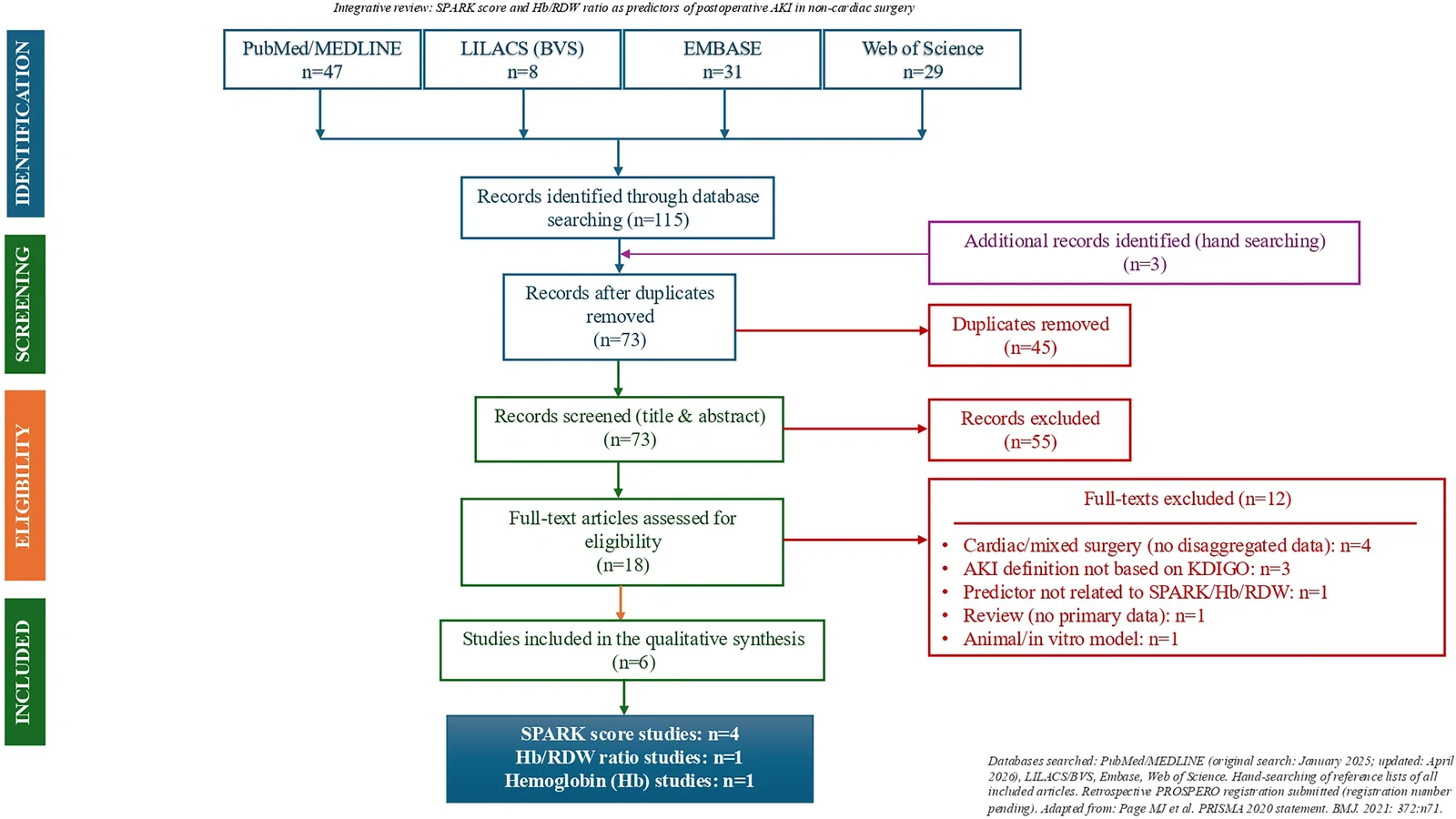
*PRISMA* 2020 flow diagram for the identification, screening, and selection of studies included in the integrative review.

Titles and abstracts of all retrieved records were independently screened by two reviewers (ACR and PMS). Full texts of potentially eligible studies were retrieved and assessed against the eligibility criteria by the same reviewers. Disagreements were resolved by consensus or through consultation with a third reviewer (MAG). The selection process was documented in the PRISMA 2020 flow diagram (Figure 1).

### Data extraction and quality assessment

Data were extracted using a standardized form, including study design, sample size, population characteristics, surgical context, incidence of AKI, operationalization of the predictor (cutoff points, timing of assessment), and performance metrics (AUC, sensitivity, specificity, and C-index). The quality of the included observational studies was assessed using the Newcastle-Ottawa Scale (NOS).

## RESULTS

The systematic search across the four databases retrieved 115 records (PubMed: n = 47; LILACS: n = 8; Embase: n = 31; Web of Science: n = 29), with an additional three records identified through manual reference screening, totaling 118 records. After removal of duplicates (n = 45), 73 records were screened by title and abstract. Of these, 18 were selected for full-text review, resulting in six studies included in the qualitative synthesis. The detailed selection process is shown in the PRISMA 2020 flow diagram (Figure 1), and the reasons for exclusion at the full-text stage are specified in the same flow diagram.

This reduced number of eligible studies (n = 6) is, in itself, a relevant finding of this review, reflecting a genuine scarcity of evidence on SPARK and Hb/RDW specifically in the context of non-cardiac surgery rather than a limitation of the search strategy, as confirmed by the expansion to the databases Embase and Web of Science.

Six studies published between 2019 and 2025 were included, most of which had observational cohort designs, either retrospective or prospective, conducted in different hospital settings and involving patients undergoing non-cardiac surgery. Sample sizes ranged from 307 to more than 130,000 participants, and nearly all studies used the KDIGO criteria to define AKI. The main methodological characteristics and model performance results are presented in Table 2.

**Table 2:** Methodological characteristics and diagnostic performance metrics of the studies included in the integrative review on predictors of postoperative acute kidney injury (PO-AKI) in non-cardiac surgery: the SPARK score and the hemoglobin-to-red cell distribution width ratio (Hb/RDW).

Author/year	Study design	N (sample)	AKI criterion	Predictor	Performance metrics	Results	Conclusion
Park et al. (2019)^20^	Retrospective multicenter cohort	Derivation: 51,041 Validation: 39,764	KDIGO (creatinine): ↑≥0.3 mg/dL within 48 h or ≥1.5 × baseline within 7 days.	SPARK	Sens: 68.35% Espec: 57.49% AUC: 0.72 (external validation) Cutoff: 10%	The higher the SPARK class, the greater the risk of PO-AKI. Appropriate stratification across increasing risk categories.	SPARK adequately reflects the risk of PO-AKI, its severity, and critical outcomes (death or need for dialysis).
Nishimoto et al. (2021)^23^	Retrospective cohort	5,135	KDIGO. Critical AKI: stage ≥2 or associated with death/renal replacement therapy before discharge.	SPARK	AUC: 0.69 (PO-AKI) AUC: 0.64 (critical AKI).	PO-AKI: 303 (5.9%); critical AKI: 137 (2.7%). Increasing incidence with higher SPARK scores.	SPARK confirmed the relationship between higher scores and greater risk; however, it showed suboptimal discrimination and calibration in the Japanese population.
Li et al. (2025)^24^	Prospective single-center cohort — ICU	973	KDIGO (creatinine): ↑≥26.5 µmol/L within 48 h or ≥150% of baseline within 7 days. Critical AKI: stage ≥2 or death/renal replacement therapy.	SPARK (original and modified)	Original SPARK: AUC = 0.703 Modified SPARK: AUC = 0.813 Cutoff: 10% Sens: 68.35% Espec: 57.49%	PO-AKI: 79 (8.1%); critical AKI: 14 (1.4%). Model adjustments using continuous variables significantly improved accuracy.	Modification of SPARK demonstrated improved predictive performance. Population-specific and methodological adjustments enhance its practical utility.
Kwon et al. (2025)^25^	Multicenter retrospective — derivation and external validation	138,812 (4 cohorts)	KDIGO (creatinine). Critical AKI: stage ≥2, dialysis, or death within 90 days.	Extended SPARK (+ AKI stage + malignancy)	Original SPARK: AUC 0.74 (PO-AKI); 0.78 (critical AKI) Extended model: C-index 0.74–0.86 (1 year); 0.68–0.82 (3 years)	The expanded model demonstrated excellent discrimination for predicting death or dialysis up to 3 years after surgery. An online calculator is available.	The expanded model shows good accuracy for short-, medium-, and long-term outcomes in non-cardiac surgery.
Zhou and Liu (2022)^26^	Retrospective cohort	35,631	KDIGO (creatinine and/or initiation of dialysis).	Hemoglobin (perioperative decrease)	AUC: 0.714 Thresholds: ↓>43 g/L (non-anemic); ↓>18 g/L (anemic) PO-AKI: 2,105 (5.9%)	A drop in hemoglobin above the defined thresholds is associated with a higher risk of AKI. There is a temporal correlation between hemoglobin decline and increased creatinine within the first 5 days.	Tolerance to hemoglobin decline varies among patients. Preoperative hemoglobin levels improve the clinical utility of the predictor.
Yuan et al. (2024)^21^	Retrospective cohort	307	KDIGO 2012 (creatinine): ↑≥0.3 mg/dL within 48 h or ≥1.5 × baseline within 7 days, or urine output <0.5 mL/kg/h for 6 h.	Hb/RDW	AUC: 0.714 Sens: 72.7% Espec: 70.8% Cutoff: ≤5.44	Patients with Hb/RDW ≤5.44 have an increased risk of orthopedic PO-AKI. The early postoperative index is strongly associated with renal risk.	Hb/RDW is effective in predicting PO-AKI in elderly patients with intertrochanteric fracture. It is a simple, accessible, and easy-to-use marker in clinical practice.

Abbreviations – AUC: area under the ROC curve; Hb: hemoglobin; PO-AKI: postoperative acute kidney injury; KDIGO: Kidney Disease: Improving Global Outcomes; RDW: red cell distribution width; Sens: sensitivity; Spec: specificity; RRT: renal replacement therapy; ICU: intensive care unit.

The indices evaluated were the SPARK score and the hematological biomarkers hemoglobin and the Hb/RDW ratio. Performance metrics included sensitivity, specificity, AUC (area under the ROC curve), and the C-index, depending on the model proposed in each study.

The original SPARK model, developed by Park et al.^20^ and applied in a South Korean cohort, demonstrated good discriminative ability, with a sensitivity of 68.35%, a specificity of 57.49%, and an AUC of 0.72 during external validation. The model adequately stratified patients according to the risk of postoperative AKI and severe outcomes, such as the need for dialysis or death.

In the validation conducted by Nishimoto et al.^23^, the SPARK model maintained the association between higher scores and an increased risk of AKI; however, it showed lower performance (AUC of 0.69 for PO-AKI and 0.64 for critical AKI), possibly due to demographic and clinical differences in the Japanese cohort.

Li et al.^24^, applied the model in a population of critically ill patients and observed that adjustments incorporating specific clinical variables significantly improved its accuracy, increasing the AUC from 0.703 (original model) to 0.813 (modified model).

Kwon et al.^25^ expanded the model by including the stage of PO-AKI and a history of malignancy. This enhanced version demonstrated excellent discriminative capacity (C-index ranging from 0.74 to 0.86 for one year and 0.68 to 0.82 for three years) and greater clinical applicability, in addition to enabling the estimation of medium and long-term outcomes. The authors also provided an interactive online calculator, facilitating the practical use of the score for risk stratification and postoperative follow-up.

The comparison of SPARK performance across different studies is summarized in Table 3.

**Table 3:** Comparative diagnostic performance of the SPARK score and its modified versions across validation cohorts.

Study	Country	N (sample)	Design	SPARK version	AUC – PO-AKI	Key limitation
Park et al. (2019)^20^	South Korea	90,805	Retrospective cohort – derivation + validation	Original	0.72	Derivation in a single South Korean population; categorized variables; no intraoperative data.
Nishimoto et al. (2021)^23^	Japan	5,135	Retrospective cohort – external validation	Original	0.69	Derivation in a single South Korean population; categorized variables; no intraoperative data.
Li et al. (2025)^24^	China (ICU)	973	Prospective cohort – validation + modification	Original → Modified	0.703 → 0.813*	Exclusively ICU patients; single center; limited generalizability.
Kwon et al. (2025)^25^	South Korea	138,812	Multicenter retrospective – derivation + 3 external validations	Extended (+ AKI stage + malignancy)	0.74–0.78^†^	Long-term outcomes; limited to Asian populations; additional variables required.

*AUC for the modified SPARK model (adjusted with continuous variables). ^†^AUC of the original SPARK version; the C-index of the expanded model ranged from 0.74 to 0.86 (at 1 year).

Abbreviations – AUC: area under the ROC curve; C-index: concordance index; PO-AKI: postoperative acute kidney injury; ICU: intensive care unit./*AUC for the modified SPARK model. ^†^AUC for the original SPARK; the C-index of the expanded model ranged from 0.74 to 0.86 (at 1 year).

Among the studies that analyzed laboratory parameters (Table 2), Zhou and Liu^26^ demonstrated that a marked postoperative drop in hemoglobin was strongly associated with the occurrence of AKI, especially within the first five days after surgery. The model showed an AUC of 0.714, indicating reasonable discrimination, and highlighted that tolerance to hemoglobin reduction varies among patients, making it essential to consider preoperative levels for appropriate clinical interpretation.

In turn, Yuan et al.^21^ evaluated the Hb/RDW ratio in elderly patients undergoing orthopedic surgery and found that values ≤5.44 were associated with a higher risk of PO-AKI. The model showed an AUC of 0.714, with a sensitivity of 72.7% and a specificity of 70.8%, establishing it as a simple, accessible, and effective marker for early detection of renal risk.

Overall, the analyzed studies indicate that both the SPARK score and its modified versions, as well as hematological parameters, may have clinical utility in predicting and stratifying PO-AKI in non-cardiac surgery.

## DISCUSSION

This integrative review synthesizes the available evidence on two accessible and low-cost predictors of PO-AKI in non-cardiac surgery: the SPARK score and the Hb/RDW ratio.

Despite the identification of only six eligible studies—a result that in itself demonstrates the scarcity of the evidence base in this specific area—the findings converge toward relevant conclusions: (1) SPARK shows moderate and variable predictive performance across populations, with AUC values ranging from 0.69 to 0.81 in external validations; (2) population-specificadjustments substantially improve its performance; and (3) the Hb/RDW ratio appears promising as a complementary predictor, although its evidence base in non-cardiac surgery remains limited. Taken together, these findings suggest that neither predictor alone provides a universal solution, but their integration may represent a pragmatic and accessible approach for perioperative renal risk stratification.

The SPARK index^20^ demonstrated good predictive capacity for stratifying PO-AKI risk. However, validations in different populations have shown divergent results, such as those observed in the Japanese cohort by Nishimoto et al. (2021)^23^, where the applied model showed suboptimal discrimination and calibration. The authors attributed this unsatisfactory performance to differences in the population profile, including a predominance of older patients, greater comorbidity burden, and more complex surgeries—factors that led to risk overestimation.

In contrast, Li et al.^24^ demonstrated that model recalibration using continuous variables and coefficient re-estimation in an ICU cohort increased the AUC. This substantial improvement reinforces that the variable performance of SPARK may reflect not only population differences but also intrinsic methodological limitations of the original model, such as the use of categorized variables and the absence of intraoperative factors.

Kwon et al.^25^ expanded the model differently while maintaining the operational simplicity of the original SPARK, achieving a C-index of up to 0.86 for predicting mortality or the need for dialysis within one year. The authors also provided an interactive online calculator, facilitating the practical use of the score for risk stratification and postoperative follow-up.

The divergence among findings from different validations highlights the need for careful external validation before clinical application. SPARK may be useful as a preoperative screening tool but requires contextual adaptation. The inherent heterogeneity of non-cardiac surgery—which includes orthopedic, abdominal, urological, thoracic, and vascular procedures—makes the development of a single, widely applicable model challenging^23,24^.

These limitations of SPARK support the investigation of additional biomarkers, among which hematological biomarkers stand out due to their wide clinical availability.

In this regard, several studies have highlighted the role of RDW as a robust and independent biomarker for predicting AKI in different clinical settings^27,28,29^, especially in patients with inflammation and hemodynamic instability—conditions commonly observed in the postoperative period. However, these studies were not included in the present review because they did not meet the inclusion criteria.

Hemoglobin has also been evaluated as a predictor. The study by Zhou and Liu^26^ demonstrated that a marked postoperative drop in hemoglobin constitutes a clinically relevant predictor of PO-AKI. The identification of specific thresholds for patients with and without preexisting anemia represents an important practical contribution: anemic patients have lower physiological reserves to withstand perioperative blood loss, requiring closer monitoring.

The combined evaluation of hematological markers in this review can be observed in the study by Yuan et al.^21^, the only one analyzing the Hb/RDW ratio in a specific non-cardiac surgical context. The findings support the hypothesis that integrating markers of tissue oxygenation (Hb) and erythrocyte heterogeneity/systemic inflammation (RDW) provides greater sensitivity than each component alone. In elderly patients with intertrochanteric fracture—a scenario marked by anemia, inflammation, and hemodynamic instability—the early postoperative Hb/RDW ratio functioned as an accessible indicator of renal risk.

The Hb/RDW index should be understood within a broader context of low-cost hematological predictors. Among these, the neutrophil-to-lymphocyte ratio (NLR) is the most extensively studied, with meta-analytic evidence supporting its association with adverse renal outcomes in various clinical settings, including major surgeries^30,31^. Similarly, the platelet-to-lymphocyte ratio (PLR) and the monocyte-to-lymphocyte ratio (MLR) have been investigated in surgical and critical care populations with promising results.

The Hb/RDW index differs from these predictors by integrating markers of oxygen-carrying capacity (Hb) and erythrocyte heterogeneity/systemic inflammation (RDW), rather than primarily reflecting the neutrophilic inflammatory response captured by the NLR. Whether Hb/RDW provides complementary or superior information compared to NLR in the specific context of non-cardiac surgery remains an open and clinically relevant question.

Future integrative reviews specifically designed to compare the performance of these hematological indices—including NLR, PLR, MLR, and Hb/RDW—in non-cardiac surgical populations would provide the comparative foundation necessary for evidence-based clinical adoption.

### Limitations

This review presents important methodological and substantive limitations that should be acknowledged. First, the small number of eligible studies (n = 6), despite an expanded search strategy across four databases, reflects the genuine scarcity of evidence on SPARK and Hb/RDW specifically in non-cardiac surgical populations—a research gap rather than a limitation of the search itself. Second, the substantial heterogeneity among the included studies (in population characteristics, types of surgery, outcome definitions, and predictor operationalization) precluded formal meta-analytic synthesis; the integrative descriptive approach adopted, although appropriate for the exploratory nature of the review, limits the precision of the conclusions. Third, the evidence base for the Hb/RDW ratio is particularly limited, with only one eligible study (Yuan et al.^21^; n = 307 elderly orthopedic patients), which restricts the generalizability of the findings to other surgical populations and age groups. Fourth, this review was not prospectively registered in PROSPERO (retrospective registration pending), which represents a limitation in transparency, although it does not affect the conduct of the review. Fifth, although studies on RDW in non-surgical contexts—sepsis^27^, acute respiratory distress syndrome^28^, and contrast-induced nephropathy^29^—were excluded from the main analysis because they did not meet the inclusion criteria, their findings provide relevant biological plausibility for RDW as a predictor of AKI in inflammatory and hemodynamically unstable states, conditions that are also present in the perioperative period of major surgeries. These findings cannot be directly generalized to the non-cardiac surgical context, but they reinforce the pathophysiological rationale for investigating the Hb/RDW index in the perioperative setting.

## CONCLUSION

The systematic search across four databases identified only six eligible studies, a finding that in itself highlights the critical scarcity of evidence regarding the application of the SPARK score and the Hb/RDW ratio in diverse non-cardiac surgical populations—representing a priority research gap.

The results of this review indicate that, although the SPARK score is a useful tool for preoperative screening, its applicability is limited by population and methodological variations, with better performance when adapted to local characteristics. In this context, simple and more accessible biomarkers such as hemoglobin variation and, especially, the Hb/RDW ratio demonstrate relevant predictive potential for PO-AKI, functioning as complements to SPARK.

The integration of these markers into predictive models may improve the accuracy of identifying at-risk patients, enabling early and individualized interventions.

Prospective multicenter studies, with harmonized definitions and populations, are needed to definitively validate these predictors and allow for future quantitative analyses.

## Data Availability

The data supporting the findings of this study were extracted from previously published studies and are available in the original articles cited in the references. The synthesized information in this review, including the search strategy, eligibility criteria, PRISMA flow diagram, and tables of included studies, is presented within the article. No additional primary datasets were generated or analyzed.
