# Supplementary material for: Analysis of novel predictors of acute kidney injury after non-cardiac surgery: an integrative review
Source: J Bras Nefrol. 2026 Aug 31;48(4):e20250347. doi: 10.1590/2175-8239-JBN-2025-0347en (PMC13544379; doi:10.1590/2175-8239-JBN-2025-0347en)
Supplement: Translation

# Translation: Análise de novos preditores de injúria renal aguda após cirurgias não cardíacas: uma revisão integrativa

**DOI:** 10.1590/2175-8239-JBN-2025-0347pt

**Authors:** Anna Carolina de Rizzo Cantoni Rosa, Petherson Mendonça dos Santos, Beatriz Moreira Silva, Eduardo Cantoni Rosa, Miguel Angelo Goes

**Affiliations:** 1 Universidade Santo Amaro, São Paulo, SP, Brasil.; 2 Universidade Federal de São Paulo, Escola Paulista de Medicina, Disciplina de Nefrologia, São Paulo, SP, Brasil.; 3 Faculdade Israelita de Ciências da Saúde Albert Einstein, São Paulo, SP, Brasil.

**Keywords:** Medição de Risco, Hemoglobina, Índices de Eritrócitos, Injúria Renal Aguda, Complicações Pós-Operatórias, Fatores de Risco.

## Abstract

**Introdução:**

A injúria renal aguda (IRA) é uma complicação comum no pós-operatório (IRA-PO), ocorrendo em até 35% das cirurgias não cardíacas. Alguns biomarcadores têm demonstrado valor na identificação precoce, mas são pouco utilizados na prática clínica devido ao alto custo. O escore SPARK e a razão hemoglobina/amplitude de distribuição eritrocitária (Hb/RDW) têm emergido como alternativas acessíveis para a predição de IRA-PO.

**Objetivo:**

Avaliar os estudos sobre os preditores SPARK e biomarcadores hematológicos utilizados na estratificação de risco para IRA-PO em cirurgias não cardíacas.

**Métodos:**

Revisão integrativa conduzida em quatro bases de dados (PubMed/MEDLINE, LILACS, Embase e *Web of Science*), abrangendo publicações entre 2019 e 2025, em português e inglês. Foram incluídos estudos com adultos submetidos a cirurgias não cardíacas. A seleção foi documentada por meio do fluxograma PRISMA 2020.

**Resultados:**

Seis estudos foram incluídos. O escore SPARK apresentou AUCs variando de 0,69 a 0,81 nas validações externas, com desempenho superior em modelos modificados para populações específicas. O índice Hb/RDW demonstrou boa acurácia (AUC 0,714; sensibilidade 72,7%; especificidade 70,8%) em pacientes idosos ortopédicos, com queda associada ao risco aumentado de IRA-PO.

**Discussão:**

A eficácia do SPARK depende do perfil populacional, destacando a importância da validação externa e de ajustes contextuais. A Hb, o RDW e a razão Hb/RDW mostram-se ferramentas promissoras como complemento ao SPARK.

**Conclusão:**

A aplicação do SPARK pode ser limitada em diferentes contextos. Biomarcadores simples como Hb, RDW e Hb/RDW apresentam bom potencial preditivo, podendo melhorar a identificação precoce de pacientes com risco de IRA-PO.

## INTRODUÇÃO

A injúria renal aguda (IRA) é uma complicação frequente no contexto cirúrgico, caracterizada por uma redução abrupta da função renal ao longo de horas ou dias, sendo diagnosticada com base no aumento dos níveis séricos de creatinina ou na redução do débito urinário, conforme os critérios do KDIGO^1^. Sua ocorrência está associada a um aumento significativo da morbidade e mortalidade, prolongamento do tempo de internação hospitalar e maior risco de progressão para doença renal crônica (DRC)^2,3,4^.

A IRA pós-operatória (IRA-PO) corresponde a até um terço dos casos de IRA em pacientes hospitalizados^5^, variando conforme o tipo de cirurgia e as condições clínicas pré-existentes dos pacientes. É mais frequente após cirurgias cardíacas; porém, em cirurgias não cardíacas, pode ocorrer em até 30–35% dos pacientes^6,7,8^.

Apesar dos avanços na compreensão da fisiopatologia da IRA-PO, ainda não existem terapias específicas capazes de reverter a disfunção renal já estabelecida, o que destaca a importância da prevenção e do diagnóstico precoce para mitigar ou evitar sua ocorrência^2^.

Nesse sentido, a detecção precoce pode reduzir a necessidade de terapia de substituição renal, bem como o tempo de internação hospitalar, a mortalidade e os custos em saúde^9,10,11^. No entanto, a detecção da IRA-PO permanece um desafio clínico, uma vez que biomarcadores tradicionais, como creatinina sérica e débito urinário, apresentam limitações importantes. A creatinina pode levar dias para refletir a deterioração da função renal, enquanto a oligúria pode ser influenciada por fatores hemodinâmicos transitórios^9^.

Dentro desse contexto, novos biomarcadores vêm sendo estudados como alternativas para a identificação precoce da lesão renal. Marcadores como a lipocalina associada à gelatinase de neutrófilos (NGAL), a molécula de lesão renal-1 (KIM-1) e os marcadores de estresse celular TIMP-2 e IGFBP-7 (NephroCheck^®^) demonstraram valor preditivo para a ocorrência de IRA-PO^12^. O uso desses biomarcadores pode aprimorar a estratificação de risco e orientar intervenções nefroprotetoras precoces (*bundles*)^13^, reduzindo potencialmente a progressão da IRA e a necessidade de terapia de substituição renal^9,14,15,16^.

Embora a literatura recomende o uso de biomarcadores em diferentes estágios da IRA, especialmente durante a fase subclínica de lesão renal, na prática clínica eles ainda não são amplamente adotados devido às limitações técnicas e aos custos associados^17,18^.

Paralelamente, escores de risco baseados em fatores perioperatórios têm sido amplamente utilizados em cirurgias cardíacas para orientar estratégias nefroprotetoras desde o período pré-operatório^19^. Por outro lado, ferramentas práticas e bem validadas para a avaliação de risco de IRA em cirurgias não cardíacas ainda são escassas.

Para suprir essa lacuna, o escore SPARK (*Simple Postoperative AKI Risk*) foi desenvolvido por pesquisadores da Coreia do Sul com o objetivo de prever IRA-PO em cirurgias não cardíacas, combinando variáveis clínicas e laboratoriais para aprimorar a estratificação de risco^20^.

O escore foi derivado de um estudo de grande coorte e baseia-se exclusivamente em dados pré-operatórios. São considerados onze fatores: idade, sexo, taxa de filtração glomerular estimada (TFGe), albuminúria (detectada por fita reagente), duração estimada da cirurgia, urgência do procedimento, presença de diabetes mellitus, uso de bloqueadores do sistema renina-angiotensina-aldosterona (SRAA), hipoalbuminemia, anemia e hiponatremia. Cada variável recebe uma pontuação proporcional à sua relevância estatística, e a soma total classifica o paciente em quatro categorias de risco. Além de prever a ocorrência de IRA, o escore SPARK também avalia a gravidade da lesão, incluindo desfechos como necessidade de diálise ou óbito em até 90 dias após a cirurgia. Sua aplicação prática permite a identificação precoce de pacientes que podem se beneficiar de monitoramento intensivo e de estratégias preventivas, tornando-se uma ferramenta promissora, especialmente quando biomarcadores não estão disponíveis^2^.

Além disso, marcadores hematológicos mais acessíveis, como a hemoglobina (Hb), a amplitude de distribuição dos eritrócitos (RDW) e a razão entre ambos (Hb/RDW), têm se mostrado promissores como preditores de IRA-PO, sugerindo que alterações na oxigenação tecidual e no estado inflamatório desempenham papel importante na fisiopatologia da lesão renal^21^.

Diante desse cenário, essa revisão integrativa tem como objetivo avaliar estudos sobre o preditor SPARK e marcadores derivados do hemograma completo na predição de IRA-PO em pacientes submetidos a cirurgias não cardíacas, analisando sua aplicabilidade clínica e identificando lacunas na evidência disponível.

## MÉTODOS

### Delineamento do estudo

Trata-se de uma revisão integrativa da literatura, que permite a inclusão de estudos com delineamentos heterogêneos, possibilitando uma síntese abrangente das evidências disponíveis sobre um determinado tema.

O presente manuscrito integra um projeto de pesquisa mais amplo intitulado “SPARK como preditor de injúria renal aguda perioperatória em cirurgias não cardíacas”. Esse projeto tem como objetivo identificar preditores de IRA perioperatória, com foco em fatores clínicos e demográficos. O estudo recebeu aprovação do Comitê de Ética em Pesquisa (CAAE: 67500523.1.3001.5553, agosto de 2023).

### Registro

Por se tratar de uma revisão integrativa, não foi realizado registro prospectivo em plataformas como o PROSPERO, uma vez que esses registros são destinados principalmente a revisões sistemáticas. Ainda assim, o rigor metodológico foi assegurado por meio de uma estratégia de busca estruturada, de critérios de elegibilidade previamente definidos e da seleção independente dos estudos.

O registro retrospectivo foi submetido ao PROSPERO (número de registro: pendente de atribuição no momento da submissão). O protocolo estará publicamente disponível após confirmação do registro.

### Pergunta de pesquisa – estrutura PICO

A pergunta de pesquisa foi formulada utilizando a estrutura PICO (População, Índice/Intervenção, Comparação, Desfechos): *“Em pacientes adultos (≥18 anos) submetidos a cirurgias não cardíacas, o escore SPARK e a razão Hb/RDW demonstram desempenho preditivo adequado para IRA-PO, medido por AUC, sensibilidade e/ou especificidade contra desfechos definidos pelos critérios KDIGO?”*, conforme apresentado na Tabela 1.

**Tabela 1:** Elemento PICO utilizado para estruturar a pergunta de pesquisa.

PICO	Operacionalização
*Population* **(População)**	Pacientes adultos (≥18 anos) submetidos a cirurgias não cardíacas eletivas ou de urgência, em qualquer cenário clínico.
*Index Test* **(Índice)**	Escore SPARK aplicado no pré-operatório e/ou razão Hb/RDW aplicada no período perioperatório ou no pós-operatório precoce.
*Comparison* **(Comparação)**	Nenhum comparador obrigatório. Quando disponível: SPARK original vs. modificado; SPARK vs. Hb/RDW; preditor vs. avaliação clínica padrão.
*Outcomes* **(Desfechos)**	Primário: IRA-PO definida pelos critérios KDIGO. Secundários: IRA crítica (KDIGO ≥2), necessidade de terapia renal substitutiva, mortalidade hospitalar ou em 90 dias.

### Estratégia de busca

A estratégia de busca foi conduzida de acordo com recomendações baseadas no checklist PRISMA^22^, utilizando quatro bases de dados eletrônicas: PubMed/MEDLINE, LILACS (via Biblioteca Virtual em Saúde — BVS), Embase e *Web of Science*. As buscas originais foram realizadas em janeiro de 2025, e uma atualização complementar foi conduzida em abril de 2026, após recomendação dos revisores. Não foi aplicada restrição de idioma na busca atualizada. A lista de referências de todos os estudos incluídos foi rastreada manualmente para identificar artigos adicionais potencialmente elegíveis.

#### PubMed/MEDLINE – *string* de busca completa:

*((“Acute Kidney Injury”[MeSH] OR “acute kidney injury”[tiab] OR “AKI”[tiab] OR “renal failure, acute”[MeSH]) AND (“Noncardiac Surgical Procedures”[MeSH] OR “non-cardiac surgery”[tiab] OR “noncardiac surgery”[tiab] OR “postoperative complications”[MeSH] OR “postoperative AKI”[tiab] OR “surgical patients”[tiab]) AND (“SPARK”[tiab] OR “Simple Postoperative AKI Risk”[tiab] OR “Hemoglobins”[MeSH Terms] OR “hemoglobin”[tiab] OR “Erythrocyte Indices”[MeSH Terms] OR “red cell distribution width”[tiab] OR “RDW”[tiab] OR “Hb/RDW”[tiab] OR “HB/RDW ratio”[tiab]))*

Filtros aplicados: publicações entre 2019 e 2025 (busca original); sem restrição de data (busca atualizada).

#### LILACS/BVS – *string* de busca completa

*(injúria renal aguda OR acute kidney injury OR AKI OR lesão renal aguda) AND (cirurgia não cardíaca OR non-cardiac surgery OR noncardiac surgery) AND (SPARK OR hemoglobina OR hemoglobin OR hemoglobins OR RDW OR Hb/RDW OR Hb/RDW ratio OR distribuição de glóbulos vermelhos OR red cell distribution width OR erythrocyte indices)*

#### Embase – *string* de busca completa

*(‘acute kidney injury’/exp OR ‘acute kidney injury’:ti,ab,kw) AND (‘non-cardiac surgery’:ti,ab,kw OR ‘noncardiac surgery’:ti,ab,kw OR ‘postoperative complication’/exp) AND (‘SPARK score’:ti,ab,kw OR ‘hemoglobin’/exp OR ‘hemoglobins’/exp OR ‘erythrocyte indices’/exp OR ‘red cell distribution width’:ti,ab,kw OR ‘RDW’:ti,ab,kw OR ‘Hb/RDW’:ti,ab,kw)*

#### Web of science – *string* de busca completa

TS = (“acute kidney injury” OR “AKI”) AND TS = (“non-cardiac surgery” OR “noncardiac surgery” OR “postoperative”) AND TS = (“SPARK” OR “hemoglobin” OR “hemoglobins” OR “red cell distribution width” OR “RDW” OR “Hb/RDW” OR “erythrocyte indices”)

### Descritores controlados (MeSH/DeCS)

Os seguintes descritores controlados foram utilizados: “Acute Kidney Injury” [MeSH]; “Hemoglobins” [MeSH: D006454]; “Erythrocyte Indices” [MeSH: D004910]; “Postoperative Complications” [MeSH]; “Noncardiac Surgical Procedures” [MeSH]. O índice Hb/RDW não constitui um descritor MeSH direto — trata-se de uma razão calculada a partir de dois descritores estabelecidos: “Hemoglobins” (D006454) e “Erythrocyte Indices” (D004910), conforme reconhecido nas diretrizes de indexação médica. Os operadores booleanos “AND” e “OR” foram utilizados de forma combinada para refinar os resultados.

### Critérios de elegibilidade

Foram incluídos estudos que atenderam a todos os seguintes critérios: (1) publicados entre 2019 e 2025 (justificativa: o escore SPARK foi publicado em 2019, sendo esse o marco inicial do tema); (2) realizados em populações humanas; (3) envolvendo participantes adultos (≥18 anos); (4) cirurgias não cardíacas; (5) preditor de interesse: escore SPARK e/ou razão Hb/RDW (ou seus componentes: Hb e RDW); e (6) desfecho: IRA-PO definida pelos critérios KDIGO.

Foram excluídos estudos que: (1) avaliaram exclusivamente cirurgias cardíacas ou populações mistas sem dados desagregados para cirurgias não cardíacas; (2) utilizaram critérios de definição de IRA distintos do KDIGO sem possibilidade de conversão; (3) eram artigos não originais (editoriais, cartas, artigos de revisão sem dados primários); ou (4) envolviam modelos animais ou estudos *in vitro*.

### Seleção dos estudos

Títulos e resumos de todos os registros recuperados foram selecionados de forma independente por dois revisores (ACR e PMS). Os textos completos dos estudos potencialmente elegíveis foram recuperados e avaliados de acordo com os critérios de elegibilidade pelos mesmos revisores. As discordâncias foram resolvidas por consenso ou por um terceiro revisor (MAG). O processo de seleção foi documentado no fluxograma PRISMA 2020 (Figura 1).

**Figura 1:** Fluxograma PRISMA 2020 do processo de identificação, triagem e seleção dos estudos incluídos na revisão integrativa.

### Extração de dados e avaliação da qualidade

Os dados foram extraídos por meio de um formulário padronizado, incluindo: delineamento do estudo, tamanho amostral, características da população, contexto cirúrgico, incidência de IRA, operacionalização do preditor (pontos de corte, momento da avaliação) e métricas de desempenho (AUC, sensibilidade, especificidade, índice C). A avaliação da qualidade dos estudos observacionais incluídos foi realizada utilizando a *Newcastle-Ottawa Scale* (NOS).

## RESULTADOS

A busca sistemática nas quatro bases de dados recuperou 115 registros (PubMed: n = 47; LILACS: n = 8; Embase: n = 31; *Web of Science*: n = 29), acrescidos de 3 registros identificados por rastreamento manual de referências, totalizando 118 registros. Após a remoção de duplicatas (n = 45), 73 registros foram triados por título e resumo. Destes, 18 foram selecionados para leitura completa, resultando em 6 estudos incluídos na síntese qualitativa. O detalhamento do processo de seleção está representado no fluxograma PRISMA 2020 (Figura 1) e os motivos para exclusão na etapa de leitura do texto completo são especificados no mesmo fluxograma.

Este número reduzido de estudos elegíveis (n = 6) constitui, por si só, um achado relevante desta revisão, refletindo a escassez genuína de evidências sobre o escore SPARK e a razão Hb/RDW especificamente no contexto de cirurgias não cardíacas — e não uma falha da estratégia de busca, conforme confirmado pela expansão para as bases de dados Embase e *Web of Science*.

Foram incluídos seis estudos publicados entre 2019 e 2025, com delineamentos majoritariamente observacionais e de coorte, retrospectivos ou prospectivos. Os estudos foram realizados em diferentes contextos hospitalares e envolveram populações submetidas a cirurgias não cardíacas. As amostras variaram de 307 a mais de 130 mil participantes, e quase todos os estudos utilizaram os critérios do KDIGO para definição de IRA. As principais características metodológicas e os resultados de desempenho dos modelos estão apresentados na Tabela 2.

**Tabela 2:** Características metodológicas e métricas de desempenho dos estudos incluídos na revisão integrativa sobre preditores de injúria renal aguda pós-operatória (IRA-PO) em cirurgias não cardíacas: escore SPARK e razão hemoglobina/amplitude de distribuição eritrocitária (Hb/RDW).

Autor/ano	Tipo de estudo	N (amostra)	Critério de IRA	Índice avaliado	Métricas de desempenho	Resultados	Conclusão
Park et al. (2019)^20^	Coorte retrospectiva multicêntrica	Derivação: 51.041 Validação: 39.764	KDIGO (creatinina): ↑≥0,3 mg/dL em 48 h ou ≥1,5 × basal em 7 dias	SPARK	Sens: 68,35% Espec: 57,49% AUC: 0,72 (validação externa) Corte: 10%	Quanto maior a classe SPARK, maior o risco de IRA-PO. Estratificação adequada em faixas de risco crescentes.	O SPARK reflete adequadamente o risco de IRA-PO, sua gravidade e desfechos críticos (óbito ou necessidade de diálise).
Nishimoto et al. (2021)^23^	Coorte retrospectiva	5.135	KDIGO. IRA crítica: estágio ≥2 ou associada a óbito/TSR antes da alta hospitalar.	SPARK	AUC: 0,69 (IRA-PO) AUC: 0,64 (IRA crítica)	IRA-PO: 303 (5,9%); IRA crítica: 137 (2,7%). Incidência crescente com maior escore SPARK.	SPARK confirmou relação entre maior escore e maior risco, porém com discriminação e calibração subótimas na população japonesa.
Li et al. (2025)^24^	Coorte prospectiva de centro único (pacientes de UTI)	973	KDIGO (creatinina): ↑≥26,5 µmol/L em 48 h ou ≥150% basal em 7 dias. IRA crítica: estágio ≥2 ou óbito/TSR.	SPARK (original e modificado)	SPARK original: AUC = 0,703 SPARK modificado: AUC = 0,813 Corte: 10% Sens: 68,35% Espec: 57,49%	IRA-PO: 79 (8,1%); IRA crítica: 14 (1,4%). Ajustes do modelo com variáveis contínuas elevaram significativamente a acurácia.	Modificação do SPARK demonstrou melhor desempenho preditivo. Ajustes populacionais e metodológicos aumentam sua utilidade prática.
Kwon et al. (2025)^25^	Observacional retrospectivo multicêntrico — derivação e validação externa	138.812 (4 coortes)	KDIGO (creatinina). IRA crítica: estágio ≥2, diálise ou óbito em 90 dias.	SPARK estendido (+ estágio IRA + malignidade)	SPARK original: AUC 0,74 (IRA-PO); 0,78 (IRA crítica)	Modelo estendido: índice C 0,74–0,86 (1 ano); 0,68–0,82 (3 anos)	Modelo expandido com excelente discriminação para prever óbito ou diálise até 3 anos pós-cirurgia. Calculadora online disponível.	Modelo expandido com boa acurácia para desfechos de curto, médio e longo prazo em cirurgias não cardíacas.
Zhou and Liu (2022)^26^	Coorte retrospectiva	35.631	KDIGO (creatinina e/ou início de diálise).	Hb (queda perioperatória)	AUC: 0,714 Limiares: ↓>43 g/L (não anêmicos); ↓>18 g/L (anêmicos) IRA-PO: 2.105 (5,9%)	Queda de Hb acima dos limiares associada a maior risco de IRA. Correlação temporal entre queda de Hb e aumento da creatinina nos primeiros 5 dias.	Tolerância à queda de Hb varia entre pacientes. Níveis pré-operatórios de Hb melhoram a utilidade clínica do preditor.
Yuan et al. (2024)^21^	Coorte retrospectiva	307	KDIGO 2012 (creatinina): ↑≥0,3 mg/dL em 48 h ou ≥1,5 × basal em 7 dias, ou débito urinário <0,5 mL/kg/h por 6 h.	Hb/RDW	AUC: 0,714 Sens: 72,7% Espec: 70,8% Corte: ≤5,44	Pacientes com Hb/RDW ≤5,44 apresentam risco aumentado de IRA-PO ortopédica. Índice pós-operatório precoce fortemente associado ao risco renal.	Hb/RDW eficaz para predizer IRA-PO em idosos com fratura intertrocantérica. Marcador simples, acessível e de fácil uso na prática clínica.

Abreviaturas – AUC: área sob a curva ROC; Hb: hemoglobina; IRA-PO: injúria renal aguda pós-operatória; KDIGO: Kidney Disease: Improving Global Outcomes; RDW: amplitude de distribuição eritrócitária; Sens: sensibilidade; Espec: especificidade; TSR: terapia de substituição renal; UTI: unidade de terapia intensiva.

Os índices avaliados foram o escore SPARK e os biomarcadores hematológicos hemoglobina e a razão Hb/RDW. As métricas de desempenho incluíram sensibilidade, especificidade, AUC (área sob a curva ROC) e índice C, dependendo do tipo de modelo proposto em cada estudo.

O modelo original SPARK, desenvolvido por Park et al.^20^ e aplicado em uma coorte sul-coreana, demonstrou bom poder discriminatório, com sensibilidade de 68,35%, especificidade de 57,49% e AUC de 0,72 na validação externa. O modelo estratificou adequadamente os pacientes de acordo com o risco de IRA pós-operatória e desfechos graves, como necessidade de diálise ou óbito.

Na validação realizada por Nishimoto et al.^23^, o modelo SPARK manteve a relação entre escores mais elevados e maior risco de IRA, porém com desempenho inferior (AUC de 0,69 para IRA-PO e 0,64 para IRA crítica), possivelmente devido a diferenças demográficas e clínicas na coorte japonesa.

Li et al.^24^, ao aplicarem o modelo em uma população de pacientes críticos, observaram que ajustes no modelo, incorporando variáveis clínicas específicas, aumentaram significativamente sua acurácia, elevando a AUC de 0,703 (modelo original) para 0,813 (modelo modificado).

Kwon et al.^25^ ampliaram o modelo ao incluir o estágio da IRA-PO e o histórico de neoplasia. Essa versão aprimorada demonstrou excelente capacidade discriminatória (índice C entre 0,74–0,86 para um ano e 0,68–0,82 para três anos) e maior aplicabilidade clínica, além de permitir a estimativa de desfechos em médio e longo prazo. Os autores também disponibilizaram uma calculadora online interativa, facilitando o uso prático do escore na estratificação de risco e no seguimento pós-operatório.

A comparação do desempenho do SPARK entre os diferentes estudos está sistematizada na Tabela 3.

**Tabela 3:** Comparação do desempenho diagnóstico do escore SPARK e suas versões modificadas entre os estudos de validação incluídos.

Estudo	País	N (amostra)	Delineamento	Versão do SPARK	AUC – IRA-PO	Principal limitação
Park et al. (2019)^20^	Coreia do Sul	90.805	Coorte retrospectiva – derivação + validação	Original	0,72	Derivação em população sul-coreana única; variáveis categorizadas; sem dados intraoperatórios
Nishimoto et al. (2021)^23^	Japão	5.135	Coorte retrospectiva – validação externa	Original	0,69	Perfil etário e de comorbidades diferente; calibração subótima; AUC inferior à coorte original
Li et al. (2025)^24^	China (UTI)	973	Coorte prospectiva – validação + modificação	Original → Modificado	0,703 → 0,813*	Exclusividade de pacientes de UTI; centro único; generalização limitada
Kwon et al. (2025)^25^	Coreia do Sul	138.812	Coorte retrospectiva multicêntrica – derivação + 3 validações externas	Estendido (+ estágio IRA + malignidade)	0,74–0,78^†^	Desfechos de longo prazo; limitado a populações asiáticas; variáveis adicionais necessárias

*AUC do modelo SPARK modificado (ajuste com variáveis contínuas). ^†^AUC da versão original do SPARK; o índice C do modelo expandido variou de 0,74 a 0,86 (1 ano).

Abreviaturas – AUC: área sob a curva ROC; índice C: índice de concordância; IRA-PO: injúria renal aguda pós-operatória; UTI: unidade de terapia intensiva./*AUC para o modelo SPARK modificado. ^†^AUC para o SPARK original; o índice C do modelo estendido variou de 0,74 a 0,86 (em 1 ano).

Dentre os estudos que analisaram parâmetros laboratoriais (Tabela 2), Zhou e Liu^26^ demonstraram que uma queda acentuada da hemoglobina no pós-operatório associou-se fortemente à ocorrência de IRA, especialmente nos primeiros cinco dias após a cirurgia. O modelo apresentou AUC de 0,714, indicando capacidade discriminatória razoável, e evidenciou que a tolerância à redução da hemoglobina varia entre os pacientes, tornando essencial considerar os níveis pré-operatórios para uma adequada interpretação clínica.

Por sua vez, Yuan et al.^21^ avaliaram a razão Hb/RDW em pacientes idosos submetidos à cirurgia ortopédica e observaram que valores ≤5,44 estavam associados a um maior risco de IRA-PO. O modelo apresentou AUC de 0,714, com sensibilidade de 72,7% e especificidade de 70,8%, consolidando-se como um marcador simples, acessível e eficaz para a detecção precoce de risco renal.

De forma geral, os estudos analisados indicam que o escore SPARK, suas versões modificadas e os parâmetros hematológicos podem apresentar utilidade clínica na predição e estratificação da IRA-PO em cirurgias não cardíacas.

## DISCUSSÃO

Essa revisão integrativa sintetiza as evidências disponíveis sobre dois preditores acessíveis e de baixo custo para IRA-PO em cirurgias não cardíacas: o escore SPARK e a razão Hb/RDW.

A despeito da identificação de apenas seis estudos elegíveis, resultado que por si só demonstra a escassez da base de evidências nessa área específica, os achados convergem para conclusões relevantes: (1) o SPARK apresenta desempenho preditivo moderado e variável entre populações, com AUC entre 0,69 e 0,81 nas validações externas; (2) ajustes específicos para cada população melhoram substancialmente seu desempenho; e (3) a razão Hb/RDW mostra-se promissora como preditor complementar, embora sua base de evidências em cirurgias não cardíacas seja ainda limitada. Em conjunto, esses achados sugerem que nenhum dos preditores, isoladamente, oferece uma solução universal, mas que sua integração pode representar uma abordagem pragmática e acessível para a estratificação de risco renal perioperatório.

O índice SPARK^20^ demonstrou boa capacidade preditiva para estratificação de risco de IRA-PO. Entretanto, validações em populações distintas revelaram resultados divergentes, como na coorte japonesa de Nishimoto et al.^23^, na qual o modelo aplicado apresentou discriminação e calibração subótimas. Os autores atribuíram esse desempenho insatisfatório às diferenças no perfil populacional, como predominância de pacientes mais idosos, com mais comorbidades e submetidos a cirurgias mais complexas, características que levaram à superestimação do risco.

Em contraste, Li et al.^24^ demonstraram que a readequação do modelo por meio do uso de variáveis contínuas e da reestimativa de coeficientes em uma coorte de UTI elevou a AUC. Essa melhora expressiva reforça que o desempenho variável do SPARK pode refletir não apenas diferenças populacionais, mas também limitações metodológicas intrínsecas ao modelo original, como o uso de variáveis categorizadas e a ausência de fatores intraoperatórios.

Kwon et al.^25^ expandiram o modelo de forma distinta, porém mantiveram a simplicidade operacional do SPARK original, o que permitiu um índice C de até 0,86 para previsão da mortalidade e da necessidade de diálise em um ano. Os autores também disponibilizaram uma calculadora online interativa, facilitando o uso prático do escore na estratificação de risco e no seguimento pós-operatório.

A divergência entre os achados das diferentes validações destaca a necessidade de uma validação externa cuidadosa antes da aplicação clínica. O SPARK pode ser útil como ferramenta de triagem pré-operatória, mas requer adaptação contextual. A heterogeneidade inerente às cirurgias não cardíacas — que engloba procedimentos ortopédicos, abdominais, urológicos, torácicos e vasculares — dificulta o desenvolvimento de um modelo único e amplamente aplicável^23,24^.

Essas limitações do SPARK sustentam a investigação de biomarcadores adicionais e, dentre estes, destacam-se os biomarcadores hematológicos, amplamente disponíveis para uso clínico.

Nesse sentido, diversos estudos destacaram o papel da RDW como biomarcador robusto e independente na predição de IRA em diferentes cenários clínicos^27,28,29^, especialmente em pacientes com inflamação e instabilidade hemodinâmica, condições comuns no período pós-operatório. No entanto, esses estudos não foram incluídos na presente revisão, por não preencherem os critérios de inclusão.

A hemoglobina tem sido outro marcador recentemente avaliado. O estudo de Zhou e Liu^26^ demonstrou que a queda acentuada na hemoglobina pós-operatória constitui um preditor clinicamente relevante de IRA-PO. A identificação de limiares específicos para pacientes com e sem anemia prévia representa uma contribuição prática importante: pacientes anêmicos possuem menor reserva fisiológica frente às perdas sanguíneas perioperatórias, exigindo atenção redobrada.

Por sua vez, a avaliação combinada de marcadores hematológicos nesta revisão pode ser analisada no estudo de Yuan et al.^21^, única análise a avaliar a razão Hb/RDW em um contexto cirúrgico não cardíaco específico. Os achados corroboram a hipótese de que a integração entre marcadores de oxigenação tecidual (Hb) e de heterogeneidade eritrocitária/inflamação sistêmica (RDW) confere ao índice Hb/RDW maior sensibilidade do que cada componente isolado. Em pacientes idosos com fratura intertrocantérica — cenário marcado por anemia, inflamação e instabilidade hemodinâmica —, a razão Hb/RDW no pós-operatório precoce funcionou como um indicador acessível de risco renal.

O índice Hb/RDW deve ser compreendido dentro de um panorama mais amplo de preditores hematológicos de baixo custo. Entre esses, o índice neutrófilo-linfócito (NLR) é o mais estudado, com evidências meta-analíticas apoiando sua associação com desfechos renais adversos em diferentes cenários clínicos, incluindo cirurgias de grande porte^30,31^. Da mesma forma, o índice plaqueta-linfócito (PLR) e o índice monócito-linfócito (MLR) têm sido investigados em populações cirúrgicas e de cuidados intensivos, com resultados promissores.

O índice Hb/RDW difere desses preditores por integrar marcadores de capacidade de transporte de oxigênio (Hb) e de heterogeneidade eritrocitária/inflamação sistêmica (RDW), em vez de refletir primariamente a resposta inflamatória neutrofílica captada pelo NLR. Se o Hb/RDW oferece informação complementar ou superior ao NLR no contexto específico das cirurgias não cardíacas permanece uma questão aberta e clinicamente relevante.

Revisões integrativas futuras, especificamente desenhadas para comparar o desempenho desses índices hematológicos — incluindo NLR, PLR, MLR e Hb/RDW — em populações cirúrgicas não cardíacas, forneceriam a base comparativa necessária para sua adoção clínica baseada em evidências.

### Limitações

Esta revisão apresenta importantes limitações metodológicas e substantivas que devem ser reconhecidas. Primeiro, o número reduzido de estudos elegíveis (n = 6), apesar da estratégia de busca expandida em quatro bases de dados, reflete a escassez genuína de evidências sobre SPARK e Hb/RDW especificamente em populações cirúrgicas não cardíacas — uma lacuna de pesquisa, e não uma falha da estratégia de busca. Segundo, a heterogeneidade substancial entre os estudos incluídos (em características populacionais, tipos cirúrgicos, definições de desfecho e operacionalização dos preditores) inviabilizou a realização de síntese meta-analítica formal; a abordagem descritiva integrativa adotada, embora adequada ao caráter exploratório da revisão, limita a precisão das conclusões. Terceiro, a base de evidências para a razão Hb/RDW é particularmente restrita, com apenas um estudo elegível (Yuan et al.^21^; n = 307 pacientes idosos ortopédicos), o que limita a generalização das conclusões para outras populações cirúrgicas e faixas etárias. Quarto, esta revisão não foi registrada prospectivamente no PROSPERO (registro retrospectivo pendente), o que representa uma limitação de transparência, embora não afete a condução da revisão. Quinto, embora estudos sobre o RDW em contextos não cirúrgicos — sepse^27^, síndrome do desconforto respiratório agudo^28^ e nefropatia induzida por contraste^29^ — tenham sido excluídos da análise principal por não atenderem aos critérios de inclusão, seus achados fornecem plausibilidade biológica relevante para o RDW como preditor de IRA em estados inflamatórios e hemodinâmicos instáveis, características também presentes no período perioperatório de grandes cirurgias. Esses achados não podem ser diretamente generalizados para o contexto cirúrgico não cardíaco, mas reforçam o racional fisiopatológico que sustenta a investigação do índice Hb/RDW no perioperatório.

## CONCLUSÃO

A busca sistemática em quatro bases de dados identificou apenas seis estudos elegíveis, achado que, por si só, evidencia a escassez crítica de evidências sobre a aplicação do escore SPARK e da razão Hb/RDW em populações cirúrgicas não cardíacas diversas — uma lacuna de pesquisa prioritária.

Os resultados desta revisão indicam que, embora o escore SPARK seja uma ferramenta útil para triagem pré-operatória, sua aplicabilidade é limitada por variações populacionais e metodológicas, com melhor desempenho quando adaptado às características locais. Nesse cenário, biomarcadores simples e mais acessíveis como a variação da Hb e, especialmente, a razão Hb/RDW, demonstram potencial preditivo relevante para IRA-PO, funcionando como complemento ao SPARK.

A integração desses marcadores a modelos preditivos pode ampliar a acurácia na identificação de pacientes em risco, favorecendo intervenções precoces e individualizadas.

Estudos prospectivos multicêntricos, que harmonizem definições e populações, são necessários para validar definitivamente esses preditores e possibilitar análises quantitativas futuras.

## Data Availability

Os dados que dão suporte aos resultados deste estudo foram extraídos de estudos previamente publicados e estão disponíveis nos artigos originais citados nas referências. As informações sintetizadas nesta revisão, incluindo a estratégia de busca, os critérios de elegibilidade, o fluxograma PRISMA e as tabelas com os estudos incluídos, estão apresentadas no próprio artigo. Não foram gerados ou analisados conjuntos de dados primários adicionais.
